# Consumption of Polyphenol-Rich *Zingiber Zerumbet* Rhizome Extracts Protects against the Breakdown of the Blood-Retinal Barrier and Retinal Inflammation Induced by Diabetes

**DOI:** 10.3390/nu7095369

**Published:** 2015-09-15

**Authors:** Thing-Fong Tzeng, Tang-Yao Hong, Yu-Cheng Tzeng, Shorong-Shii Liou, I-Min Liu

**Affiliations:** 1 Department of Pharmacy and Master Program, Tajen University, Pingtung County 90741, Taiwan, R.O.C.; E-Mails: d850084@yahoo.com.tw (T.-F.T.); ssliou@tajen.edu.tw (S.-S.L.); 2 Department of Biotechnology, Collage of Pharmacy and Health Care, Tajen University, Pingtung County 90741, Taiwan, R.O.C.; E-Mail: tyhong@tajen.edu.tw; 3 St. Dominic’s Catholic High School, Kaohsiung 80288, Taiwan, R.O.C.; E-Mail: 2770727@yahoo.com.tw

**Keywords:** diabetic retinopathy, *Zingiber zerumbet* rhizome, NF-κB, p38 MAPK

## Abstract

The present study investigates the amelioration of diabetic retinopathy (DR) by *Zingiber zerumbet* rhizome ethanol extracts (ZZRext) in streptozotocin-induced diabetic rats (STZ-diabetic rats). ZZRext contains high phenolic and flavonoid contents. STZ-diabetic rats were treated orally with ZZRext (200, 300 mg/kg per day) for three months. Blood-retinal barrier (BRB) breakdown and increased vascular permeability were found in diabetic rats, with downregulation of occludin, and claudin-5. ZZRext treatment effectively preserved the expression of occludin, and claudin-5, leading to less BRB breakdown and less vascular permeability. Retinal histopathological observation showed that the disarrangement and reduction in thickness of retinal layers were reversed in ZZRext-treated diabetic rats. Retinal gene expression of tumor necrosis factor-α, interleukin (IL)-1β, IL-6, vascular endothelial growth factor, intercellular adhesion molecule-1 and vascular cell adhesion molecule-1 were all decreased in ZZRext-treated diabetic rats. Moreover, ZZRext treatment not only inhibited the nuclear factor κB (NF-κB) activation, but also downregulated the protein expression of p38 mitogen-activated protein kinase (MAPK) in diabetic retina. In conclusion, the results suggest that the retinal protective effects of ZZRext occur through improved retinal structural change and inhibiting retinal inflammation. The antiretinopathy property of ZZRext might be related to the downregulation of p38 MAPK and NF-κB signal transduction induced by diabetes.

## 1. Introduction

The blood-retinal barrier (BRB) breakdown is the hallmark of many vascular diseases in the retina. The major component of this functional barrier has long been recognized to be at the level of the tight junctions between adjacent endothelial cells [1]. Appropriate functioning of these junctional proteins in endothelial cells is essential for the maintenance of the normal function of the retina, and decreased expression of these proteins is known to cause various vascular pathologies, including diabetic retinopathy (DR), the most common and serious complication of diabetes mellitus (DM). It has been found that DR occurs in both type 1 and type 2 diabetes, and that nearly all case of type 1 and type 2 diabetes lead to DR after prolonged duration [2]. Various complications associated with DR have been noted, and these include vitreous hemorrhage, retinal detachment and glaucoma [3].

The increased circulating levels of glucose accumulate in the retinal endothelial cells and result in the activation of various biochemical pathways. A number of signaling mechanisms, like nuclear factor kappa-B (NF-κB), mitogen-activated protein kinase (MAPK), intracellular adhesion molecules (ICAM)-1, and interleukin (IL)-1β, have been found to be implicated in the pathogenesis of DR [4]. Although the Diabetes Control and Complications Trial and United Kingdom Prospective Diabetes Study show that intensive glycemic control is associated with a lower risk of retinopathy compared to conventional therapy in type 1 and type 2 diabetes, achievement and maintenance of glycemic control remains difficult or impossible in many patients [5]. Laser photocoagulation is currently the primary method of treatment for patients with diabetic retinopathy who are at a high risk of vision loss, but unfortunately this is not always effective for improving vision [6,7]. Considering the limitations and side effects of current treatments of DR, a concomitant treatment with some other drugs that prevent or delay the development of micro- and macrovascular complications in diabetes seems to be required.

*Zingiber zerumbet* (L) Smith, a wild ginger belonging to the tropical and subtropical family of Zingiberaceae, is well known as *Hong qiu jiang* in Taiwan. The plant is widely cultivated in village gardens in the tropics for its medicinal properties and as a marketable spice [8]. The chemical constituents that are more frequently found in *Zingiber zerumbet* rhizomes (ZZR) are flavonoids, such as kaempferol, quercetin, and curcumin [9,10]. The volatile oils of ZZR have been reported to contain a cyclic sesquiterpene zerumbone or 2,6,9-humulatrien-8-one as the major component, as well as humulene and camphene [9,10]. In addition to its regular use as a food flavoring and appetizer, ZZR has also been used traditionally for medicinal purposes, *i.e.* as a folk remedy for headaches, swellings, colds, ulcers, sores and loss of appetite, nausea and even menstrual discomfort in Asian, Indian, Chinese, and Arabic cultures since ancient times [10,11]. Furthermore, the methanol extract of ZZR possesses inhibitory effects on platelet-activating factor and Den2 virus NS2B/NS3 protease activity [12,13]. A recent study has shown that ZZR ethanol extracts (ZZRext) exert a potential blood glucose lowering effect in diabetic rats [14]. The beneficial impact of ZZRext on insulin sensitivity has also been demonstrated [15]. In addition, ZZRext has been shown to possess efficacy against inflammation in diabetic nephropathy [16]. ZZRext is valued for its ability to promote glucose homeostasis, and it may therefore be utilized as an adjuvant therapy in the control of diabetes-related microvascular complications. However, the possibility that ZZRext could prove beneficial in the amelioration of DR has not yet been explored.

The observation that streptozotocin (STZ)-induced diabetic rats develop retinal lesions similar to those observed in humans with diabetes has attracted widespread attention to this animal model of human DR [17]. Based on this, the present study is designed to observe the amelioration of ZZRext on STZ-induced DR in rats and its underlying mechanism.

## 2. Materials and Methods

### 2.1. Plant Material and Extraction

ZZR was purchased from a local market in Dongshan, Dongshan Dist. (Tainan City, Taiwan) during September 2014. Macroscopic and microscopic examinations, as well as thin-layer chromatography and high-performance liquid chromatography, were used to confirm the authenticity of the plant material provided. Random amplified polymorphic DNA analysis of ZZR supplied was also performed to identify DNA polymorphisms. The voucher specimen (Lot No.20140923) was deposited in our laboratory. A coarse powder of ZZR was obtained after comminution and filtration (20–40 mesh), and 20 g powder was ground in a 95% (v/v) ethanol solution using a mixer, followed by extraction of the samples for three days with vigorous shaking. The filtrate was then isolated by membrane filtration for removal of the macro- and micro-molecular components. The extraction yield from dry weight of ZZRext was 12.3%. ZZRext was concentrated using rotary-vacuum evaporation at 50 °C, and then freeze-dried.

### 2.2. Measurement of Total Phenolic Content

The total phenolic content of ZZRext was determined using a spectrophotometer according to the Folin-Ciocalteu colorimetric method [18]. The total phenolic content of ZZRext was expressed as mg gallic acid (Sigma-Aldrich, Inc., Saint Louis, MI, USA) equivalents (GAE)/g.

### 2.3. Measurement of Total Flavonoids

The total flavonoid content was determined as previously described [19], with slight modifications. Briefly, 0.25 mL of ZZRext (100 μg/mL) was added to a tube containing 1 mL of double-distilled water. Next, 0.075 mL of 5% NaNO2, 0.075 mL of 10% AlCl3, and 0.5 mL of 1 mmol/L NaOH were added sequentially at 0, 5, and 6 min. Finally, the volume of the reacting solution was adjusted to 2.5 mL with double-distilled water. The solution had an absorbance of 410 nm. The results were expressed in mg quercetin (Sigma-Aldrich, Inc., St. Louis, MO, USA) equivalents (QE)/g.

### 2.4. Generation of Diabetic Rat Model

Male Wistar rats (8–10 weeks of age, 200–250 g) were obtained from the National Laboratory Animal Center (Tainan City, Taiwan). To induce diabetes the rats were given a single intravenous injection of 60 mg/kg streptozotocin (STZ; Sigma-Aldrich, Inc.). After 1 week, rats with non-fasting blood glucose levels >350 mg/dL, polyuria, and glucosuria were defined as diabetic and used for the experiments. All animal procedures were performed according to the Guidelines for the Care and Use of Laboratory Animals of the National Institutes of Health (United States), as well as the guidelines of the Animal Welfare Act. These studies were conducted with the approval of the Institutional Animal Care and Use Committee (IACUC) at Tajen University (approval number: IACUC 103-10; approval date: 12 October 2014).

### 2.5. Treatment Protocols

At two weeks after the injection of STZ, a group of eight rats was dosed by oral gavage once per day for three consecutive months either with ZZRext doses of 200 or 300 mg/kg in a volume of 1.5 mL/kg distilled water. The selection of dosage regime for the present study was based on a previous study which reported that ZZRext at 200 and 300 mg/kg had potential effects in improving insulin resistance in diabetic rats [15]. Another group of STZ-diabetic rats (*n* = 8) was treated orally for three months with calcium dobesilate (CaD; purity ≥99.0%, Sigma-Aldrich, Inc.) at the daily dose of 500 mg/kg, a therapeutic agent for prevention of DR [20], and these rats were used as a positive control group. A vehicle-treated group of STZ-diabetic rats (*n* = 8) and a group of normal rats (*n* = 8) were treated with 1.5 mL/kg distilled water only over the same treatment period. The body weight, plasma glucose and glycosylated hemoglobin (HbA_1c_) levels were monitored during the whole experimental process. The diagnostic kit for determination of the plasma levels of glucose (Cat. No. COD12503) was purchased from BioSystem (Barcelona, Spain). Commercial enzyme-linked immunosorbent assay (ELISA) kits were used to quantify HbA_1c_ levels (Integrated Bio Ltd., Taipei, Taiwan; Cat. No. CSB-E08140r). All analyses were performed in accordance with the instructions provided by the manufacturers.

At the end of the experiments, the animals were fasted overnight (18 h) and then anesthetized using an intraperitoneal injection of sodium pentobarbital (60 mg/kg). Blood was collected directly from the abdominal aorta of each animal. Rat eyes from each group were removed. For biochemical analysis, eyes were flash frozen in liquid nitrogen and stored at −80 °C, while for histological analysis eyes were formalin fixed. Each group with eight rats was used to study each analysis.

### 2.6. Measurement of BRB Permeability

Retinal endothelial permeability was measured using the Evans blue (EB) dye injection method, as previously described, but with minor modifications [21]. Briefly, EB (Sigma-Aldrich, Inc.) was dissolved in saline (30 mg/mL), filtered, and injected through the tail vein at a dosage of 45 mg/kg within 10 s. After the dye had circulated for two hours, the rats were anesthetized with pentobarbital (40 mg/kg body weight), the chest cavity was opened, and cardiac perfusion was performed via the left ventricle with 1% paraformaldehyde in citrate buffer (0.05 mol/L, pH 3.5) under a constant pressure of 120 mmHg. Immediately after perfusion, the retinas were carefully dissected under an operating microscope. After the retinas were fully dried at 4 °C they were weighed, and EB dye was then extracted by incubating each sample in 150 μL formamide for 18 h at 70 °C. Absorbance was measured using 100 μL of the supernatant at 620 nm and 740 nm. The concentration of EB in the extracts was calculated from a standard curve and normalized by the weight of the dry retina.

### 2.7. Retinal Cytokines and Adhesion Molecules Determination

Retinas were lysed in ice-cold RIPA buffer (50 mmol/L, Tris-HCl (pH 8), 150 mmol/L NaCl, 1 mmol/L EDTA, 0.1% sodium dodecyl sulfate, 1% IGEPAL^®^ CA-630 and 0.5% sodium deoxicholate) containing protease inhibitor cocktails (Sigma-Aldrich, Inc., Saint Louis, MI, USA; Cat. No. P83490) and centrifuged for 15 min at 10,000 × *g* at 4 °C [22]. The supernatants were collected and assayed for protein content using a Bio-Rad DC protein assay kit (Bio-Rad Laboratories, Milan, Italy). ELISA kits for the determination of TNF α (Cat. No. ab46070), IL-1β (Cat. No. ab100768), IL-6 (Cat. No. ab100772), vascular endothelial growth factor (VEGF; Cat. No. ab100786), and ICAM-1 (Cat. No. ab100763) were obtained from Abcam Inc. (Cambridge, MA, USA). Vascular cell adhesion molecule 1 (VCAM-1) ELISA kit (Cat. No. NB-E30582) was obtained from Novatein Biosciences (Cambridge, MA, USA). Samples were assayed in duplicate according to the manufacturers’ instructions.

### 2.8. Light Microscopy and Morphometric Analysis

The eyeballs from eight rats per group were enucleated and cut into half by coronal section through the ora serrata. The vitreous was removed, and the posterior half of the eye was immersed in 4% paraformaldehyde for 24 h, and then sectioned, stained with haematoxylin and eosin (H&E). Ganglion cells were counted in the central and peripheral retina (both peripheral sides, nasal and temporal), and average ganglion cell number was counted per 100 mm length of the retina from each group.

The thickness of the retina, and that of the outer and inner nuclear layers, was measured in the central and peripheral retina, and the respective measurements were then averaged. All measurements were performed under a light microscope with an attached digital camera (C3040-AD6, Olympus, Tokyo, Japan) by an experienced pathologist.

### 2.9. Cytoplasmic and Nuclear Extraction

According to the manufacturer’s instructions for the nuclear extraction kit (Active Motif, CA, USA), each fresh isolated retina was homogenized in 200 μL of ice-cold hypotonic buffer (10 mmol/L NaCl, 2 mmol/L MgCl_2_, 10 mmol/L N-[2-hydroxyethyl]piperazine-N′-[2-ethanesulfonic] acid, 20% glycerol, 0.1% Triton X-100, 1 mmol/L dithiothreitol, 3 μL of 1% P-40, complete protease inhibitor cocktail, pH 7.4) for 15 min, was centrifuged at 14,000 × *g* for 10 min at 4 °C. The supernatant containing the cytoplasmic protein fraction was used for determination of protein levels on occludin, claudin-5, and ZO-1. The remaining nuclear pellet was resuspended in 50 μL of ice-cold extract buffer (hypotonic buffer, 39.8 μL of 5 mol/L NaCl and 5 μL of 10 mmol/L dithiothreitol) for 10 min and centrifuged at 14,000 × *g* for 10 min at 4 °C. The supernatant containing the nuclear fraction was used for quantification of NF-κB activity, expression and activation of p38 mitogen-activated protein kinases (p38 MAPK).

### 2.10. Western Blot Analysis

Before immunoblotting the protein concentration of each tissue was determined using a Bio-Rad protein assay kit (Bio-Rad Laboratories, Japan) and bovine serum albumin as a standard, to ensure equal loading among lanes. Cytosol (70 μg total protein) and nuclear extracts (50 μg total protein) were separated on a 7.5%–15% polyacrilamide gel and electophoretically transferred to nitrocellulose membrane. Membranes were blocked with 5% non-fat dry milk in Tris-buffered saline Tween (20 mmol/L Tris, pH 7.6, 137 mmol/L NaCl, and 0.1% Tween 20) for three hours at room temperature, followed by an overnight incubation at 4 °C with the primary antibodies occludin (Santa Cruz Biotechnology, Inc., Santa Cruz, CA, USA; Cat. No. sc-5562), claudin-5 (Santa Cruz Biotechnology, Inc., Santa Cruz, CA, USA, Cat. No. sc-28670), ZO-1 (Santa Cruz Biotechnology, Inc., Cat. No. sc-10804), p38 MAPK (Cell Signaling Technology, Inc., Beverly, CA, USA; Cat. No. 9212), phospho-p38 MAPK (Thr180/Tyr182) (Cell Signaling Technology, Inc., Cat. No. 9211), or β-actin (Santa Cruz Biotechnology, Inc.; Cat. No. sc-130656). The level of lamin A (Santa Cruz Biotechnology, Inc., Santa Cruz, CA, USA; Cat. No. sc-20680) was estimated for equal loading of the nuclear sample. All antibodies were used at a dilution of 1:1000. After washing three times with Tris-buffered saline Tween 20 (TBST), incubation with appropriate horseradish peroxidase-conjugated secondary antibodies was performed for one hour at room temperature. After three additional TBST washes, the immunoreactive bands were visualized by enhanced chemiluminescence (Amersham Biosciences, Buckinghamshire, UK) according to the manufacturer’s instructions. Band densities were determined using ATTO Densitograph Software (ATTO Corporation, Tokyo, Japan) and quantified as the ratio to β-actin. The mean value for samples from the vehicle-treated normal rats on each immunoblot, expressed in densitometry units, was adjusted to a value of 1.0. All experimental sample values were then expressed relative to this adjusted mean value. Tissue sections were sampled from four independent experiments.

### 2.11. Real-Time Polymerase Chain Reaction (PCR)

Total RNA was extracted from rat retinas using Trizol reagent (Invitrogen; Boston, MA, USA) according to the manufacturer’s protocol. RNA was quantified by A_260_ and its integrity verified by agarose gel electrophoresis using ethidium bromide for visualization. For the reverse transcriptase reaction, 1 μg of total RNA per sample and 8.5 μg/μL random hexamer primers were heated to 65 °C for 5 min and then quenched on ice. This mixture was combined with 500 μmol/L each of dATP, dTTP, dCTP, and dGTP, 10 mmol/L DTT, 20 mmol/L Tris-HCl (pH 8.4), 50 mmol/L KCl, 5 mmol/L MgCl_2_, 40 units of RNaseOUT™ recombinant ribonuclease inhibitor (Invitrogen) and 100 units SuperScript III reverse transcriptase (Invitrogen). Samples were subjected to DNase (Promega; Madison, WI, USA) treatment at 37 °C for 20 min in a GeneAmp 9700 Thermal Cycler (Applied Biosystems; Foster City, California, USA) and then held at 4 °C. Later, aliquots were taken for immediate use in PCR, the remainder of the cDNA was stored at −20 °C. mRNA expression was measured by quantitative real-time PCR in a fluorescent temperature Lightcycler 480 (Roche Diagnostics; Mannheim, Germany). Primers for amplification of each gene are listed in Table 1. The highly specific measurement of mRNA was carried out for TNFα, IL-1β, IL-6, VEGF, ICAM-1, VCAM-1, and β-actin using the LightCycler system (Bio-Rad). Primers were designed using Primer Express Software version 2.0 (Applied Biosystems; Foster City, CA, USA). The PCR reaction was performed using the following cycling protocol: 95 °C for 5 min, followed by 45 cycles of 95 °C for 5 s, 58 °C for 15 s, and 72 °C for 20 s. Dissociation curves were run after amplification to identify the specific PCR products. The mRNA expression levels were normalized to β-actin mRNA levels and calculated according to the delta-delta Ct method [23].

**Table 1:** Sequences of primers used for real-time Polymerase Chain Reaction (PCR) analysis in this study.

Target gene	Primers	Sequence
TNFα	FP	ACACCATGAGCACGGAAAGC
	RP	CCGCCACGAGCAGGAA
IL-1β	FP	AATGGACAGAACATAAGCCAACA
	RP	CCCAAGGCCACAGGGAT
IL-6	FP	GTTGCCTTCTTGGGACTGATG
	RP	ATACTGGTCTGTTGTGGGTGGT
VEGF	FP	ACAGGGAAGACAATGGGATGA
	RP	GGGCCAGGGATGGGTTT
ICAM-1	FP	CGGGTTTGGGCTTCTCC
	RP	GCCACTGCTCGTCCACATAG
VCAM-1	FP	ATCTTCGGAGCCTCAACGG
	RP	CCAATCTGAGCGAGCGTTT
β-actin	FP	TGTGATGGTGGGAATGGGTCAG
	RP	TTTGATGTCACGCACGATTTCC

FP, forward primer; RP, reverse primer.

### 2.12. Quantification of NF-κB Activation

NF-κB activity was determined using a TransAM® NF-κB p65 transcription factor assay kit (Active Motif) according to the procedures provided by the manufacturer. Twenty micrograms of retinal nuclear extracts were incubated with an oligonucleotide containing the NF-κB consensus site, and were then incubated with monoclonal and secondary antibodies directed against the NF-κB p65 subunit. The reaction was quantified at 450 nm.

### 2.13. Statistical Analysis

Data are expressed as the mean ± standard deviation (S.D.). Statistical analysis was performed with one-way analysis of variance (ANOVA). Dunnett range post-hoc comparisons were used to determine the source of significant differences, where appropriate. A *p*-value < 0.05 was considered statistically significant.

## 3. Results

### 3.1. Total Phenol and Flavonoid Contents of ZZRext

The total phenolic and flavonoid contents of ZZRext were 432.3 ± 10.4 mg GAE/g dry weight and 128.6 ± 8.9 mg QE/g dry weight, respectively.

### 3.2. Status of Body Weight, Blood Glucose, and Glycosylated Hemoglobin

After three months of diabetes, the weight gain in STZ-diabetic rats was significantly less when compared with normal rats, and the blood glucose levels were significantly higher (Table 2). The reduction in body weight was not obvious in STZ-diabetic rats receiving ZZRext or CaD during the experimental period. The blood glucose lowering effect was obvious when STZ-diabetic rats were treated with 200 (16.4% ± 2.9% decrease) or 300 mg/kg/day ZZRext (32.6% ± 3.4% decrease) for three months (Table 2).

Treatment with 200 or 300 mg/kg/day ZZRext for three months decreased the levels of HbA_1c_ in STZ-diabetic rats by 16.2% ± 3.1% and 27.5% ± 2.6%, respectively, relative to the value in STZ-diabetic rats that received vehicle (Table 2). The plasma levels of glucose and HbA_1c_ in STZ-diabetic rats treated with CaD were found to be similar to the values obtained for vehicle-treated diabetic animals (Table 2).

**Table 2:** Changes in the body weight, fasting blood glucose and glycosylated hemoglobin content in experimental animals at the end of the three-month treatment.

Groups	Body weight (g/rat)	Plasma glucose (mg/dL)	HbA_lc_ (%)
Normal rats			
vehicle-treated	337.1 ± 11.9	95.6 ± 5.7	4.9 ± 0.9
STZ-diabetic rats			
vehicle-treated	217.1 ± 13.9 *	423.3 ± 10.3 *	14.2 ± 1.2 *
ZZRext 200-treated	265.4 ± 10.6 *^,#^	341.4 ± 12.4 *^,#^	11.9 ± 1.0 *^,#^
ZZRext 300-treated	285.9 ± 12.7 *^,#^	285.3 ± 11.6 *^,#^	10.3 ± 0.8 *^,#^
CaD-treated	270.6 ± 14.5 *^,#^	401.2 ± 12.9 *	13.6 ± 1.3 *

STZ-diabetic rats were dosed by oral gavage once per day for three months with 200 mg/kg/day ZZR extracts (ZZRext 200), 300 mg/kg/day ZZR extracts (ZZRext 300) or 500 mg/kg calcium dobesilate (CaD). Normal or STZ-diabetic rats receiving vehicle treatment were given the same volume of distilled water. Values (mean ± Standard Deviation (S.D.)) were obtained for each group of eight animals. * *p* < 0.05 compared to the values of vehicle-treated normal rats. # *p* < 0.05 compared to the values of vehisdcle-treated STZ-diabetic rats.

### 3.3. Retinal Vascular Permeability

**Figure 1:**
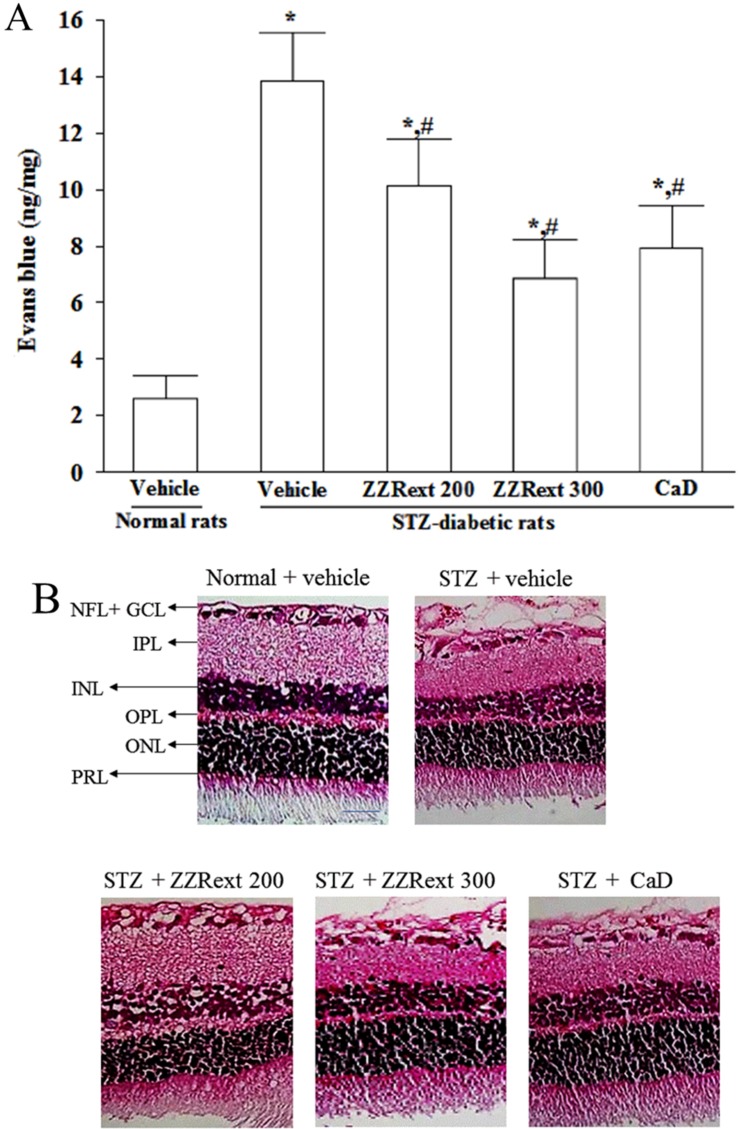

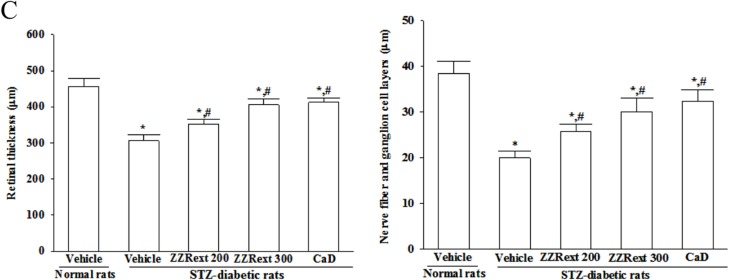
Effects of treatments on vascular permeability and retinal histology. Streptozotocin-induced (STZ)-diabetic rats were administered 200 mg/kg/day ZZRext (STZ + ZZRext 200), 300 mg/kg/day ZZRext (STZ + ZZRext 300) or 500 mg/kg calcium dobesilate (STZ + CaD) by oral gavage once daily for three months. Another group of STZ-diabetic rats (STZ + vehicle) and normal rats (normal + vehicle) were administered the same volume of vehicle. (**A**) Vascular permeability was measured with Evans blue as a tracer. (**B**) Representative images of H&E stained retinas (scale bar: 50 μm). GCL; ganglion cell layer; INL, inner nuclear layer; IPL, inner plexiform layer; NFL, nerve fiber layer; OPL, outer plexiform layer; ONL, outer nuclear layer; PRL, photoreceptor layer. (**C**) Retinal thickness and nerve fiber and ganglion cell layers were measured in each group. Values (mean ± Standard Deviation (S.D.)) were obtained from eight animals in each group. * *p* < 0.05 compared to the values of vehicle-treated normal rats. # *p* < 0.05 compared to the values of vehicle-treated STZ-diabetic rats.

Increased leakages of EB dye were observed in the retinas of STZ-diabetic rats (Figure 1A). The retinal vascular permeability in STZ-diabetic rats receiving treatments was significantly higher than normal but significantly lower than the levels seen in the diabetic rats (Figure 1A). There was also a significant decrease (42.6% ± 2.3%) in retinal vascular permeability in STZ-diabetic rats treated with CaD when compared with the vehicle-treated counterparts (Figure 1A). Treatment of STZ-diabetic rats with ZZRext for three months decreased retinal vascular permeability in a dose-dependent manner (Figure 1A).

### 3.4. Light Microscopy and Morphometric Analysis

The morphological changes in retinas of rats are shown in Figure 1B. The retinas of STZ-diabetic rats were significantly thinner than those of normal rats. After the administration of ZZRext, the retinas of STZ-diabetic rats were thicker than those of their vehicle-treated counterparts (Figure 1B,C). H&E stained retinal sections showed loss of nerve fiber and ganglion cells in diabetic retina compared to normal retina. However, ZZRext-treated diabetic retinas showed significantly higher nerve fiber and ganglion cells numbers (Figure 1B,C). Similar results were obtained for CaD-treated STZ-diabetic rats (Figure 1B,C).

### 3.5. Retinal mRNA and Protein Expressions of Tight Junction Proteins

**Figure 2:**
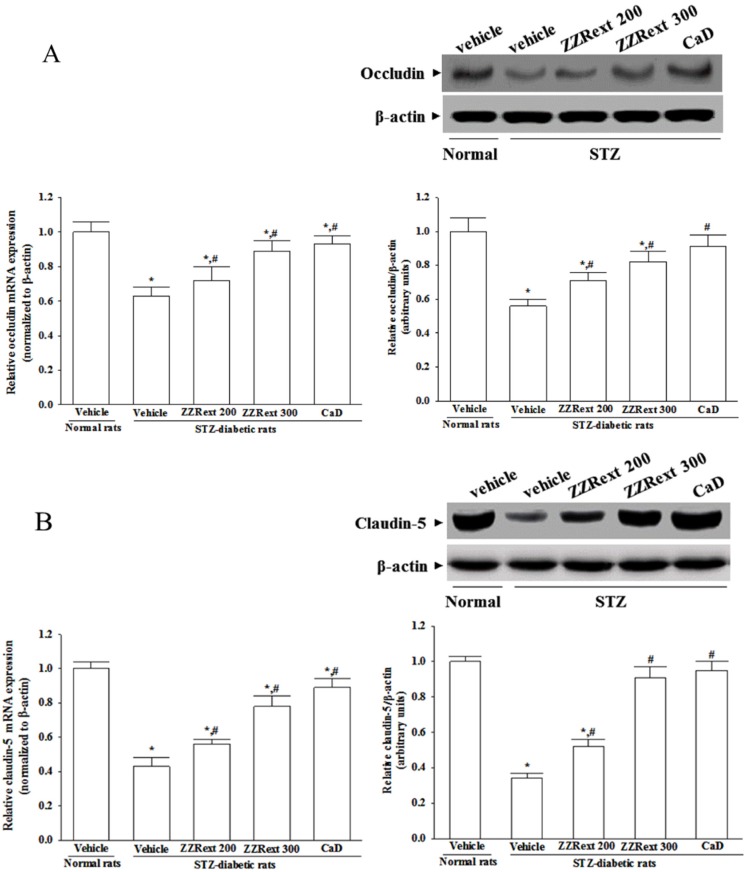

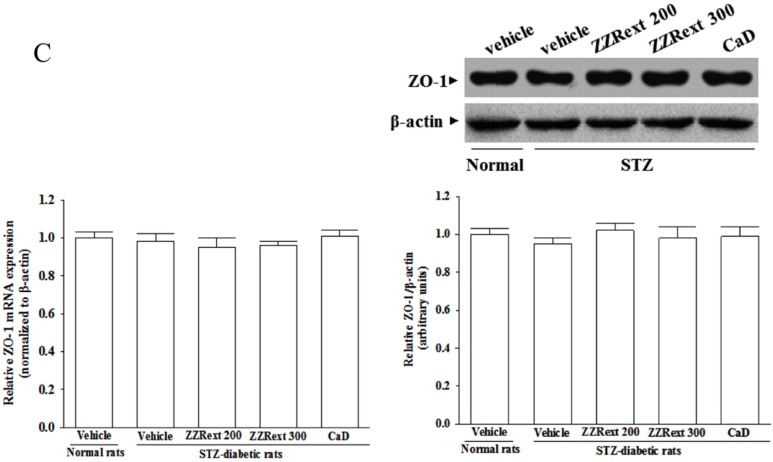
Effects of treatments on the mRNA and protein levels of (**A**) occludin, (**B**) claudin-5, and (**C**) ZO-1 in retina of rats. STZ-diabetic rats (STZ) were administered 200 mg/kg/day ZZRext (ZZRext 200), 300 mg/kg/day ZZRext (ZZRext 300) or 500 mg/kg calcium dobesilate (CaD) by oral gavage once daily for three months. Another group of STZ-diabetic rats and normal rats (normal) were administered the same volume of vehicle. Data are mean ± S.D. (standard deviation) from eight rats per group, and the experiments were repeated independently at least three times with similar results. * *p* < 0.05 compared to the values of vehicle-treated normal rats. # *p* < 0.05 compared to the values of vehicle-treated STZ-diabetic rats.

The retinal expression of occludin was significantly decreased in the STZ-diabetic rats compared with those in normal rats at both mRNA and protein levels, which were upregulated by ZZRext treatment, with an increase of 14.3% ± 2.7% and 26.8% ± 3.5%, respectively (for 200 mg/kg/day) and with an increase of 41.3% ± 2.8%, and 46.4% ± 3.9%, respectively (for 300 mg/kg/day), when compared with those observed in the vehicle-treated counterparts (Figure 2A). CaD treatment also increased retinal occludin mRNA and protein levels to 47.6% ± 5.1% and 52.5% ± 4.9%, respectively, of those invehicle-treated STZ-diabetic rats (Figure 2A).

Retinal mRNA and protein levels of claudin-5 in STZ-diabetic rats were clearly lower than those of the normal rats, and were up-regulated by ZZRext treatment: increases of 30.2% ± 2.8% and 52.9% ± 4.1%, respectively, for 200 mg/kg/day, increases of 41.2% ± 4.2% and 58.3% ± 5.1%, respectively, for 300 mg/kg/day. Similar results were obtained in the CaD-treated STZ-diabetic rats (Figure 2B).

No significant changes were observed in the retinal mRNA and protein levels of ZO-1 among STZ-diabetic rats compared to the normal group (Figure 2C). Treatment of STZ-diabetic rats with ZZRext or CaD did not appear to affect the ZO-1 expression (Figure 2C).

### 3.6. Retinal mRNA and Protein Expressions of Inflammatory Cytokines and Adhesion Molecules

**Figure 3:**
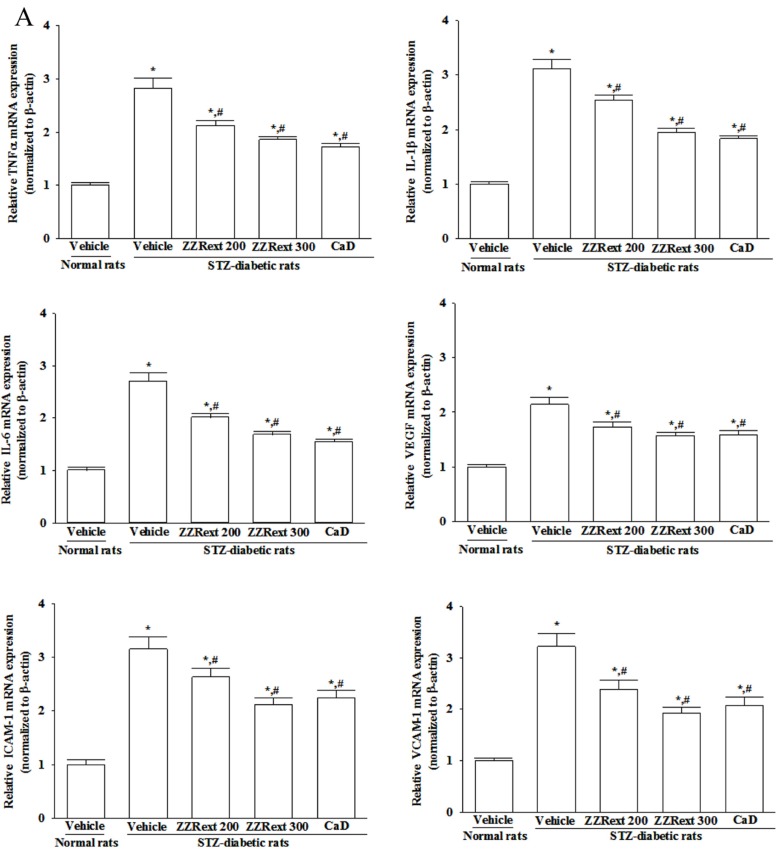

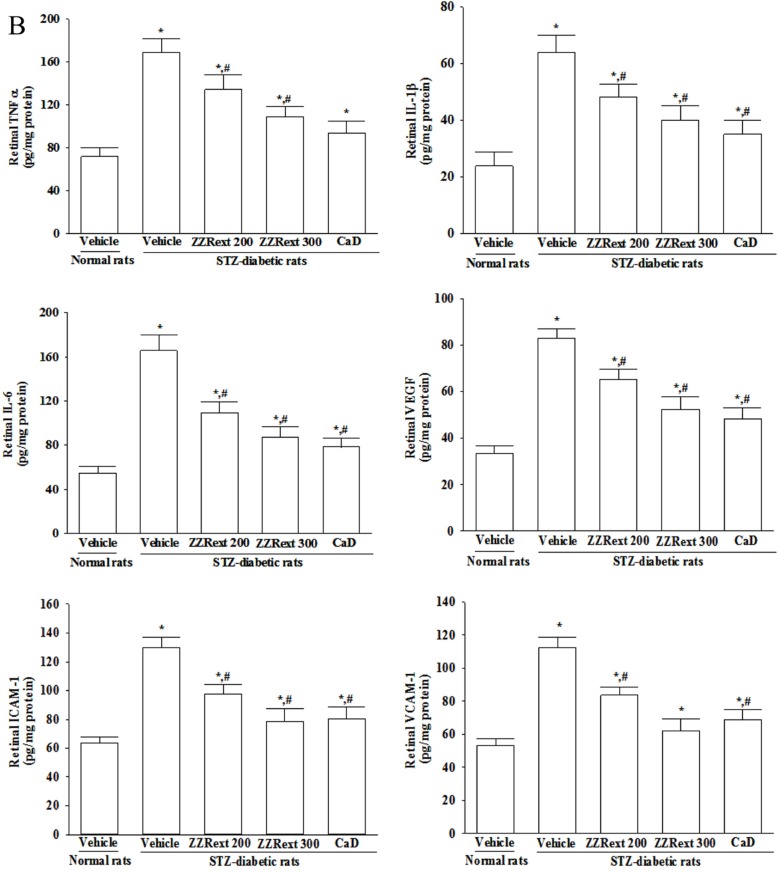
Effects of the treatments on the (**A**) mRNA and (**B**) protein levels of inflammatory cytokines and adhesion molecules in retina of rats. STZ-diabetic rats were administered 200 mg/kg/day ZZRext (ZZRext 200), 300 mg/kg/day ZZRext (ZZRext 300) or 500 mg/kg calcium dobesilate (CaD) by oral gavage once daily for three months. Another group of STZ-diabetic rats and normal rats were administered the same volume of vehicle. Data are mean ± S.D. (Standard Deviation) from eight rats per group, and the experiments were repeated independently at least three times with similar results. * *p* < 0.05 compared to the values of vehicle-treated normal rats. # *p* < 0.05 compared to the values of vehicle-treated STZ-diabetic rats.

STZ-diabetic rats had higher retinal mRNA and protein levels of TNF-α, IL-1, IL-6 and VEGF as compared to those of the control rats (Figure 3). Both the mRNA and protein levels of retinal cytokines in ZZRext (200 and 300 mg/kg/day)-treated STZ-diabetic rats were lower than those of their vehicle-treated counterparts (Figure 3). CaD treatment also reduced retinal mRNA and protein levels of TNF-α, IL-1, IL-6 and VEGF in STZ-diabetic rats, as compared to those of their vehicle-treated counterparts (Figure 3). The treatment with the highest dose (300 mg/kg) of ZZRext produced the same effect as CaD treatment (Figure 3).

STZ-diabetic rats had greater retinal mRNA and protein levels of ICAM-1 and VCAM-1 compared to the normal group (Figure 3). Treatment of STZ-diabetic rats with ZZRext induced dose-dependent reductions in retinal ICAM-1 and VCAM-1 mRNA expression (Figure 3A). The retinal protein levels of ICAM-1 and VCAM-1 in STZ-diabetic rats receiving ZZRext treatment were also decreased in a dose-dependent manner (Figure 3). The treatment with the highest dose (300 mg/kg) of ZZRext produced the same effect as CaD treatment (Figure 3).

### 3.7. The Activation of p38 MAPK in Retina

**Figure 4:**
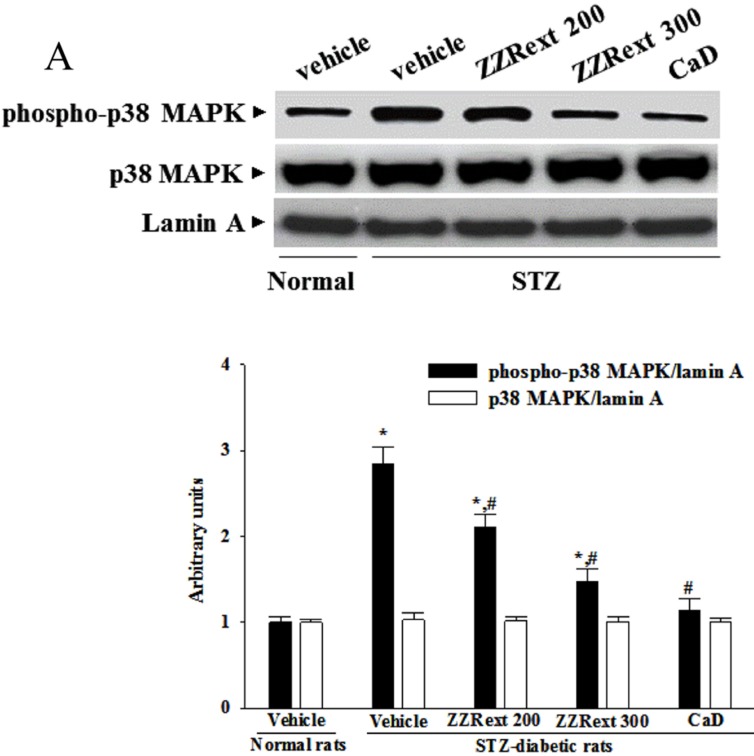

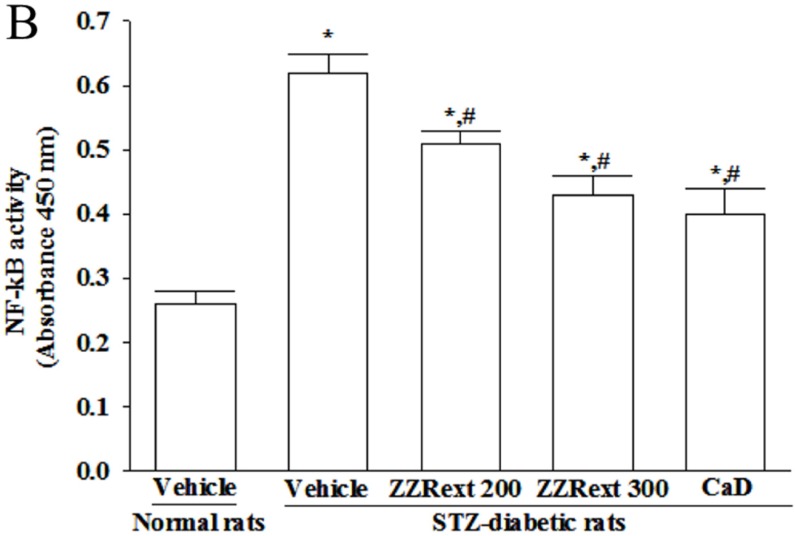
Effects of the treatments on diabetes induced retinal p38 MAPK and NF-κB activation. STZ-diabetic rats (STZ) were administered 200 mg/kg/day ZZRext (ZZRext 200), 300 mg/kg/day ZZRext (ZZRext 300) or 500 mg/kg calcium dobesilate (CaD) by oral gavage once daily for three months. Another group of STZ-diabetic rats and normal rats (normal) were administered the same volume. (**A**) The activation of p38 MAPK was determined by Western blotting, analyzing the phospho-p38/p38 MAPK ratio. (**B**) NF-κB activation was determined in retinal homogenates by ELISA using an antibody specific for the p65 subunit of NF-κB. Data are mean ± S.D. (Standard Deviation) from eight rats per group, and the experiments were repeated independently at least three times with similar results. * *p* < 0.05 compared to the values of vehicle-treated normal rats. # *p* < 0.05 compared to the values of vehicle-treated STZ-diabetic rats.

The immunoblot results showed that the degree of phosphorylation of p38 MAPK was 2.9-fold higher in the retina of STZ-diabetic rats as compared to the normal group (Figure 4A). These STZ-induced up-regulations in the degree of phosphorylation of p38 MAPK were reversed in the retina after three months of treatment with ZZRext in a dosedependent manner (Figure 4A). Similar results were obtained for CaD-treated STZ-diabetic rats (Figure 4A). No alteration in total retinal p38 MAPK contents was found among the various groups (Figure 4A).

### 3.8. The NF-κB Activity in Retina

STZ caused a 2.4-fold increase in retinal NF-κB activity relative to the level seen in the normal group (Figure 4B). The STZ-induced upregulation of NF-κB activity was reduced by 17.7% ± 4.6% and 30.6 ± 5.7% relative to that in STZ-diabetic rats after three months of treatment with 200 or 300 mg/kg/day ZZRext, respectively (Figure 4B). The retinal NF-κB activity was significantly reduced (by 35.2% ± 3.7%) in CaD-treated STZ-diabetic rats compared with the vehicle-treated counterparts (Figure 4B).

## 4. Discussion

DR causes breakdown of the BRB, resulting in vascular leakage and subsequent macular edema, which is a major cause of visual loss in DR. Like the effects of CaD, ZZRext treatment significantly decreased retinal vascular permeability. The main effect of CaD is to reduce microvascular permeability, leading to improved visual acuity [24]. The results suggest that ZZRext has distinct protective effects on STZ induced microvessel injury in the rat model of diabetes. Loss of BRB might also lead to compositional changes to the extracellular fluid in the retina, resulting in neuronal cell loss, especially in the ganglion cell layer [25]. The results of the H&E evaluation showed that the total retinal thickness and the combined nerve fiber and ganglion cell layers were maintained in the ZZR-treated STZ-diabetic rats compared with the untreated diabetic rats. Prevention of structural disorganization of the retina in diabetic rats may be the beneficial action of ZZRext in preventing DR.

The major component of BRB has long been recognized to be at the level of the tight junctions between adjacent endothelial cells [1]. Occludin and claudins are responsible for the direct cell-to-cell attachment in the tight junction barrier, and are a crucial determinant of tight junction permeability properties in endothelial cells [26,27]. Claudin-5 is found specifically in endothelial cells, and may be involved in the establishment of the barrier function [28]. The zonula occluden proteins (ZO-1, -2, and -3) coordinate the assembly of the junctional complex and enable interaction with components of the cytoskeleton, which is also important for BRB functioning. Diabetes decreases the amount of occludin and claudin-5, but not the content of ZO-1 [29,30]. ZZRext prevented alterations in tight junction proteins, indicating that its protective effects against the increase in BRB permeability are due to its stabilizing effects on these. In fact, the activation of p38 MAPK has been reported in the retinas of diabetic rats, and is associated with BRB breakdown [31]. Similar to the effect of CaD, treatment with ZZRext reduced the elevated levels of p38 MAPK in the retinas of STZ-diabetic rats, suggesting that the protective effects of ZZRext against the BRB breakdown induced by diabetes might be related to the modulation of p38 MAPK signal transduction.

Epidemiological studies and clinical trials strongly support the notion that hyperglycemia is the principal cause of microvascular and macrovascular complications [32]. Therefore, effective blood glucose control is the key to preventing or reversing diabetic complications and improving the quality of life in diabetic patients. The reductions in diabetes-induced hyperglycemia and HbA1c level found in this work after three months of ZZRext treatment are in line with previous research [16]. The concentration of HbA_1c_ is considered as a good marker for diagnosis and prognosis of diabetes complications [33]. Different to the effects of CaD, our results suggest that the retinal protective effect of ZZRext might be related to plasma glucose lowering pathway(s), and indicate that ZZRext has beneficial effects in attenuating the microvascular complications of diabetes, including DR.

There is increasing evidence that inflammatory processes have a considerable role in the pathogenesis of DR, with multiple studies showing an association of various systemic as well as local inflammatory factors and the progression of DR. Extensive research has verified the potential role of inflammatory mediators in DR. TNF, IL-1, and IL-6 are common proinflammatory cytokines, and these are increased in serum, vitreous, or retinas from diabetic patients or rats [34,35]. VEGF has been reported to have a role in neovascularizaton and increased permeability [36]. In addition, increases in adhesion molecules, such as ICAM-1 and VCAM-1, have been found to be related to the progression of DR [37]. ZZRext has been shown inhibit proinflammatory factors, chemokines, or adhesion molecules expression in the kidneys of STZ-diabetic rats [16]. In the current work we confirmed that ZZRext suppressed the gene expression of a series of proinflammatory cytokines, which may result in decreased adhesion molecule expression in the retinas of STZ-diabetic rats. Although good glycemic control remains the best and most widely accepted means of inhibiting diabetic complications, inhibition of inflammation might help inhibit retinopathy, even in the presence of hyperglycemia [38]. The combined antihyperglycemic and anti-inflammatory actions of ZZRext should be particularly advantageous, and may work synergistically in preventing diabetic-induced retinal damage.

NF-κB activation is known to mediate the expression of cytokines and adhesion molecules [39]. Diabetes has been shown to activate NF-kB in rodent retinas, and to cause migration of the p65 subunit into the nuclei of retinal endothelial cells, pericytes, ganglion cells, or cells of the inner nuclear layer [40,41]. DNA-binding experiments have demonstrated NF-κB to be activated in retinal endothelial cells or pericytes exposed to elevated glucose concentration in the retinas of diabetic rats [42]. The inhibition of NF-κB might be a critical step for the prevention of cascading inflammatory responses in DR. Under our experimental conditions, ZZRext inhibited retinal NF-κB activation in STZ-diabetic rats, as evidenced by a decrease in NF-κB activity. These results suggest that ZZRext’s protection against DR may also occur partly by acting against diabetes-induced retinal inflammation in the NF-κB-dependent pathway.

Phenolic compounds from fruits and vegetables are receiving increasing attention from consumers and manufacturers as a result of epidemiological studies suggesting an association between the consumption of polyphenol-rich foods or beverages and the prevention of certain chronic diseases [43]. ZZRext has high levels of polyphenolic flavonoids, and its pharmacological effect in ameliorating DR may be attributed to the presence of these. The specific components of ZZRext that are mainly responsible for its protective effects on retinas will be identified in future research work.

## 5. Conclusions

The antiretinopathy property of ZZRext was confirmed in this study by the increased number of neurons in the ganglion cell layer and thickness of the total retina and retinal nuclear layer in diabetic rats. These findings also provided novel evidence that ZZRext reversed the activation of p38 MAPK, and reduced tight junction protein impairment, ultimately acting against the BRB breakdown in the retinas of diabetic rats. Dampening NF-κB activation, and thereby limiting the inflammatory response, has also been suggested as a possible underlying mechanism of ZZRext’s effect in preventing DR. ZZRext is thus a potential functional food to protect against DR.
